# *Post-hoc* Analysis of Pharmacodynamics and Single-Agent Activity of CD3xCD123 Bispecific Antibody APVO436 in Relapsed/Refractory AML and MDS Resistant to HMA or Venetoclax Plus HMA

**DOI:** 10.3389/fonc.2021.806243

**Published:** 2022-01-13

**Authors:** Justin Watts, Tara L. Lin, Alice Mims, Prapti Patel, Cynthia Lee, Anoush Shahidzadeh, Paul Shami, Elizabeth Cull, Christopher R. Cogle, Eunice Wang, Fatih M. Uckun

**Affiliations:** ^1^ Sylvester Comprehensive Cancer Center, University of Miami, Miami, FL, United States; ^2^ Cancer Center and Medical Pavillon, University of Kansas, Westwood, KS, United States; ^3^ Wexner Medical Center/James Cancer Hospital, The Ohio State University, Columbus, OH, United States; ^4^ Harold C. Simmons Comprehensive Cancer Center, Department of Internal Medicine, Division of Hematology-Oncology, University of Texas Southwestern Medical Center, Dallas, TX, United States; ^5^ Department of Regulatory Affairs and Clinical Research, Aptevo Therapeutics, Seattle, WA, United States; ^6^ Huntsman Cancer Institute, University of Utah, Salt Lake City, UT, United States; ^7^ Greenville Health System, Institute for Translational Oncology Research, Greenville, SC, United States; ^8^ Department of Medicine, Division of Hematology & Oncology, University of Florida, Gainesville, FL, United States; ^9^ Roswell Park Comprehensive Cancer Center, Department of Medicine, Buffalo, NY, United States; ^10^ Immuno-Oncology Program, Ares Pharmaceuticals, St. Paul, MN, United States

**Keywords:** AML – acute myeloid leukaemia, CD123 expression, venetoclax (BCL2 inhibitor), bispecific antibody (bsAb), myelodysplastic and myeloproliferative syndromes

## Abstract

APVO436 is a recombinant bispecific antibody designed to direct host cytotoxic T-cells to CD123-expressing blast cells in patients with hematologic malignancies. APVO436 showed promising tolerability and single-agent activity in relapsed or refractory (R/R) acute myeloid leukemia (AML) and myelodysplastic syndrome (MDS). The primary purpose of this *post-hoc* analysis was to evaluate the therapeutic and pharmacodynamic effects of APVO436 in 14 R/R AML/MDS patients who had failed treatment with hypomethylating agents (HMA) or venetoclax plus HMA prior to being enrolled in the APVO436 Phase 1 dose-escalation study that was recently completed. Eight of these 14 patients had R/R AML and had failed treatment with HMA (N=2) or venetoclax plus HMA (N=6). The remaining 6 patients had R/R MDS and had also failed treatment with HMA (N=5) or venetoclax plus HMA (N=1). They were treated with APVO436 at submicrogram dose levels >0.08 mcg/kg that were active in preclinical NOD/SCID mouse xenograft models of AML. APVO436 activated patients’ T-cells as evidenced by reduced numbers of circulating CD123^+^CD34^+^ and CD33^+^CD34^+^ peripheral blasts. Single-agent activity was observed at dose levels ranging from 0.1 mcg/kg to 0.7 mcg/kg in 4 R/R AML patients (50%), including 3 patients with prolonged stable disease (SD) and one patient with complete remission (CR). Likewise, 3 MDS patients had SD (50%) and 3 additional MDS patients (50%) had a marrow CR at dose levels ranging from 0.1 mcg/kg to 0.8 mcg/kg. The median survival for the combined group of 14 R/R AML/MDS patients was 282 days. This early evidence of single-agent activity of APVO436 in R/R AML/MDS patients who failed HMA with or without venetoclax provides proof of concept supporting its *in vivo* immunomodulatory and anti-leukemic activity and warrants further investigation of its clinical impact potential.

## Introduction

AML is a common hematologic malignancy in adults with more than 10 thousand anticipated deaths in the US alone for 2021 (SEER Program, www.seer.cancer.gov). Notably, older patients with newly diagnosed AML respond poorly to standard induction chemotherapy. Therefore, there is an urgent and unmet need for effective new treatment modalities for elderly patients with newly diagnosed AML (1–6). In recent years, a number of promising treatment regimens have been identified for this high-risk AML patient population, such as the combination of venetoclax with hypomethylating agents (HMA) (1, 7–9). By comparison, no effective salvage treatment strategies exist for elderly patients with relapsed AML who often suffer cumulative organ toxicity from previous chemotherapy representing an additional hurdle to identifying effective options for their reinduction therapy (10, 11). Not surprisingly, these patients have a dismal prognosis and are in urgent need of new strategies for their chemo-resistant leukemia.

As a member of the BCL-2 homology 3 (BH3)-mimetic class of compounds, Venetoclax can disrupt the association of the proapoptotic BH3-only proteins such as BIM and BID with the antiapoptotic protein B-cell lymphoma 2 (BCL-2) (12, 13). In addition, BCL-2 inhibition by Venetoclax has the potential to selectively damage chemotherapy-resistant leukemic stem cell populations (LSCs) by inhibition of amino acid metabolism and reduction of oxidative phosphorylation (14, 15). Notably, the combined use of venetoclax and a hypomethylating agent (HMA) such as azacitidine (AZA) reduced oxidative phosphorylation in AML cells and killed primary LSC populations obtained from AML patients (15).

AML-associated CD123 antigen is the α-chain of IL-3 receptor, and it is broadly expressed on AML cells, including the LSC populations (16–18). Its expression is associated with chemotherapy resistance and poor prognosis (16, 19, 20). Several biotherapeutic agents targeting CD123 have been developed as biotherapeutic agents against AML, including the CD123-directed recombinant human IL3 fusion toxin Tagraxofusb (SL-401) and bispecific antibodies targeting CD123 antigen, such as bispecific T-cell engagers, dual affinity retargeting antibodies, bispecific killer cell engagers, and trispecific killer cell engagers (21–25). APVO436 is a humanized bispecific antibody designed to direct host cytotoxic T-cells (CTLs) to CD123-expressing blast cells from patients with hematologic malignancies (25–28).

APVO436 showed promising tolerability and single-agent activity in relapsed or refractory (R/R) AML and MDS (28, 29). The primary purpose of this *post-hoc* analysis was to evaluate the therapeutic and pharmacodynamic effects of APVO436 in 14 R/R AML/MDS patients who had failed treatment with HMA or venetoclax plus HMA prior to being enrolled in the APVO436 Phase 1 study.

## Materials and Methods

### APVO436

APVO436 is a humanized bispecific antibody (BiAB) that targets CD123 and CD3ϵ (25–27).

### Clinical Study

The primary study was a multi-institutional Phase 1B clinical dose-escalation trial of APVO436 in patients with relapsed/refractory AML and higher-risk myelodysplastic syndrome (MDS) (ClinicalTrials.gov identifier: NCT03647800). APVO436 exhibited a promising tolerability and manageable treatment-emergent AEs (28, 29). The weekly target dose levels for cohorts 2–10 were 1 mcg for Cohort 2, 3 mcg for Cohort 3, 9 mcg for Cohort 4, 18 mcg for Cohort 6A, 12 mcg for Cohort 6B, 24 mcg for Cohort 7, 36 mcg for Cohort 8, 48 mcg for Cohort 9, and 60 mcg for Cohort 10 (28). The exploratory studies included pre-planned evaluation of the pharmacodynamic effects of APVO436 on flow cytometrically quantitated CD123^+^ target cells in peripheral blood samples in relationship to clinical responses. The analysis of the single-agent activity of APVO436 in R/R AML/MDS patients who failed HMA with or without venetoclax was a *post-hoc* analysis that was not planned as the success of the Venetoclax + HMA regimen could not be predicted in 2018 when the Phase 1B trial of APVO436 was initiated. Likewise, the enrollment of an adequate number of patients for such an analysis could not be anticipated or estimated in advance. 

### Patient Characteristics

Eight patients, including 3 males and 5 females with a median age of 66 years (Mean ± SE = 65 ± 6 years) of whom 7 were Caucasian and 1 was African American, had R/R AML (**Table 1**). 4 males and 2 females had R/R MDS. They had a median age of 75 years (Mean ± SE = 75 ± 2 years) of whom 5 were Caucasian and 1 was Asian, had R/R MDS. 5 of 8 AML patients and all 6 MDS patients were ≥60 years of age (**Table 1**). Of the 8 AML patients, 4 had AML with MDS-related features – one of these patients also had FLT3-ITD gene mutation -, 1 had AML with recurrent genetic abnormalities, 2 had AML with gene mutations and 1 had AML-NOS (M0-AML) (**Table 1**). All 6 MDS patients had MDS with excess blasts according to WHO classification (MDS-EB-1 or MDS-EB-2). Five of these patients had IPSS prognosis scores consistent with an intermediate-1 (IM-1) or intermediate-2 (IM-2) risk group and one had high-risk MDS (**Table 1**). Patient characteristics and treatment outcome data are shown in **Table 1**.

**Table 1:** Patient Characteristics and Treatment Outcomes.

Parameter	Number of patients
**Age**	
≥60 years	AML: 5; MDS: 6
<60 years	AML: 3; MDS: 0
**Gender**	
Male	AML: 3; MDS: 4
Female	AML: 5; MDS: 2
**Race**	
Caucasian	AML: 7; MDS: 5
Black	AML: 1; MDS: 0
Asian	AML: 0; MDS: 1
**Adverse Events**	
DLT	AML: 0; MDS: 0
Grade ≥3 AE	AML: 2***; MDS: 1^$^
CRS	AML: 2 (Grade 1-2); MDS: 1 (Grade 1)
	
AML**	8 (UPN01-UPN08)
AML with MDS features	4
AML with recurrent genetic abnormalities	1
AML-NOS	1
AML with gene mutations	2
	
**MDS^&&^**	6 (UPN09-UPN14)
MDS-EB-1	5
MDS-EB-2	1
	
**Previous Therapy**	
Failed HMA without Venetoclax	7 (AML: 2; MDS: 5)
Failed HMA plus Venetoclax	7 (AML:6; MDS: 1)
	
**Best Overall Response (BOR)**	
**AML (N=8)**	
SD	5
CR	1
PD	2
TTP ≥90 days	5 (Median: 211 days, Range: 38->224 days)
**MDS (N=6)**	
SD (includes marrow CR)	6
Marrow CR	3
TTP (≥90 days	6 (Median: 189 days, Range: 104-321 days)

^**^The karyotypes/genetic mutations were: UPN01: -7, del(5q)/TP53; UPN02: -7, del(5q),del(17p)/TP53; UPN03: inv(16)/ND;UPN04: t(1;15),t(5;10),11q23/FLT-ITD; UPN05: 46,XX,del(20)(q11.2q13.1)/ND; UPN06: 46,XX/ND; UPN07: t(2;15)/NF1,RUNX1,GATA2,IKZF1; UPN08: -Y/FLT3-ITD.

^&&^UPN09: IPSS Score: 1.5; Risk classification: Intermediate-2; Anemia (Hgb 9.7 g/dL), Thrombocytopenia (Plt 30,000/µL), Interrmediate risk karyotype, 47,XX+8, Multiple genetic mutations (NRAS, ASXL-1, PTPNII, RUNX1, STAG2), progressed to CMML-MPN with an absolute monocyte count 17,600/µL around Cycle 10

UPN10: IPSS score: 1.0; Risk classification: Intermediate-1; Anemia (Hgb 8.0 g/dL), thrombocytopenia (Plt: 9,000/µL), severe neutropenia (ANC: 0.29x10^3^/µL), 7.7% blasts in bone marrow, cytogenetics not available. Achieved a marrow CR with bone marrow myeloblast count of 2.4%.

UPN11: IPSS score: 2.5; Risk classification: High Anemia (Hgb 9.3 g/dL, thrombocytopenia (Plt: 23,000/µL) (- non-severe neutropenia with ANC 1.3 x10^3^/µL), intermediate risk cytogenetics with MDS related aberrations 11q- and -7, 11.3% blasts in the bone marrow. Achieved a marrow CR with bone marrow myeloblast countdown to 0% at C2D1.

UPN12: IPSS score: 0.5; Risk classification: Intermediate-1No pancytopenia (Hgb 11 g/dL, Plt: 100,000/µL, ANC: 1.1 x10^3^/µL). Favorable karyotype: 46, XY, 8.2% blasts in bone marrow. Achieved a marrow CR with bone marrow myeloblast count reduced to 2% at C2D1.

UPN13: IPSS score: 2.0; IPSS score: 1.0; Risk classification: Intermediate-1; Anemia (Hgb 7.2 g/dL), thrombocytopenia (Plt: 8,000/µL), severe neutropenia (ANC: 0.5x10^3^/µL), 5% blasts in bone marrow, complex karyotype (poor risk category): 46,XY,der(12;19)(q10;p10), +mar[8]/46,XY, del(20)(q11.2q13.1). Intermediate risk karyotype with 12(p) aberration on cytogenetics. 5% blasts in bone marrow.

UPN14: IPSS score: 1.0, Risk classification: Intermediate-1. Anemia (Hgb 9.3/dL), Intermediate risk karyotype with t(1;2)(p36.3;p21) on cytogenetics, 6% blasts in bone marrow.

^***^Grade ≥3 Adverse events and Any Grade CRS/Neurotoxicity: One AML patient, UPN05, had Grade 3 sepsis, Grade 3 diarrhea, Grade 3 vomiting, and Grade 1 CRS and Grade 1 neurotoxicity. Another AML patient, UPN06, had Grade 3 confusion as well as Grade 2 CRS.

^$^One MDS patient, UPN09, had multiple transient Grade 3-4 AEs that have resolved; She had tumor lysis syndrome (TLS) Grade 3 on C1D2, lasting 2 days; TLS Grade 3 on C4D1 lasting 2 days; Anemia, Grade 3 on C5D1 lasting 8 days, Anemia Grade 3 on C7D22 lasting 8 days, Anemia Grade 3 on C6D22 lasting 12 days, Anemia Grade 3 on C7D8 lasting 4 days; decreased platelet count on C4D15 Grade 3 lasting 7 days, decreased platelet count Grade 4 on C4D22 lasting 50 days, decreased platelet count Grade 3 on C6D15 lasting 2 days, decreased platelet count Grade 4 on C6D22 lasting 33 days; hyperglycemia Grade 3 on C5D5 lasting 2 days.

IPSS, International Prognosis Scoring System; 2016 WHO myelodysplastic syndrome subtypes, MDS with excess blasts (MDS-EB).

No DLT or Grade 5 AE was observed in any of the 14 cases analyzed. Among the 8 AML patients, 6 had no CRS, one patient had Grade 1 CRS lasting 2 days, and one patient had transient Grade 2 CRS lasting 2 days (**Table 1**). There were 2 SAEs: One SAE was reported for UPN05 who developed sepsis, diarrhea and vomiting on C6D5 showing full recovery within 5 days. Grade 2 CRS of UPN06 was also reported as an SAE due to hospitalization but lasted only 2 days and fully resolved (**Table 1**). No SAEs were reported for any of the 6 MDS patients. One MDS patient experienced Grade 1 CRS on C2D1 lasting one day and another MDS patient experienced transient several Grade 3-4 AEs, including tumor lysis syndrome (TLS), Anemia, decreased platelet count, and hyperglycemia (**Table 1**).

### Ethics Statement and Study Approval

The study protocol was approved by a Central IRB (WCG; IRB00000533) as well as the local IRBs of the investigative sites. Each patient provided a written informed consent (ICF) prior to enrollment.

### Flow Cytometry

Immunophenotyping was performed on cryopreserved peripheral blood mononuclear cells from patients by standard flow cytometry using a BD LSR II flow cytometer and FACSDiva Software Version 8.0.2 fluorochrome-labeled monoclonal antibodies reactive with CD5 (anti-human CD5, clone REA782 [PE-Vio770), CD45 (anti-human CD45, Clone H130, V500, BD Biosciences #560777), CD34 (anti-human CD34, Clone REA1164, VioBright 515, Miltenyl Biotech #130-120-517), CD38 (anti-human CD38, clone HIT-2, BV605, Biolegend#303532), and CD123 (anti-human CD123, Clone 9F5, AF647, BD Biosciences #563599) antigens.

### Statistical Analyses

Standard statistical methods and the GraphPad Prism 9 statistical program (GraphPad Software, LLC, San Diego, CA) were used in data analysis. Survival data was analyzed by the Kaplan-Meier method (28–30).

## Results

### Pharmacodynamic Effects of APVO436

**Figure 1:**
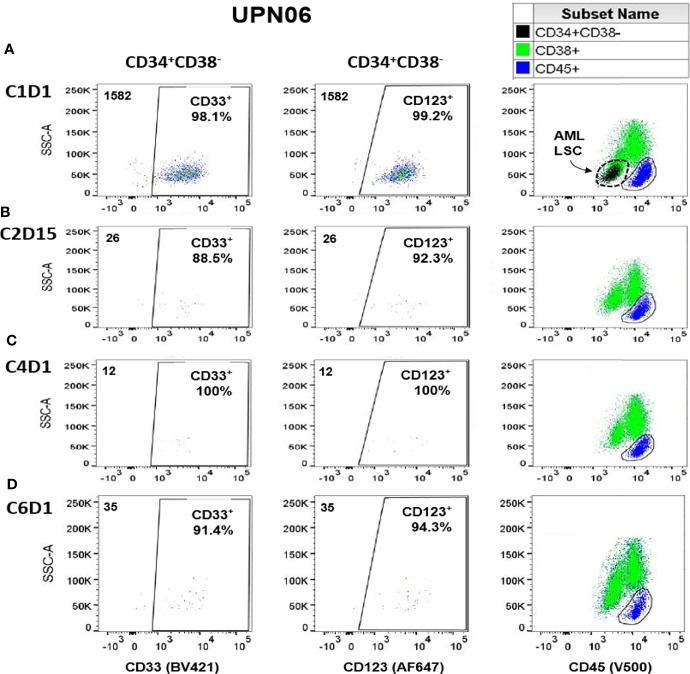
Depletion of Circulating CD123^+^CD34^+^CD38^-^ and CD33^+^CD34^+^CD38^-^ Cells in VENAZA-resistant Relapsed AML Patient Receiving APVO436 Monotherapy. The numbers in the left upper corner in the first 2 columns represent the total CD34^+^CD38^-^ cell numbers of which the vast majority co-expressed both CD123 and CD33. Panels **(A–D)** show the results at specific study time points: **(A)** C1D1 = Cycle 1, Day 1; **(B)** C2D15 = Cycle 2, Day 15; **(C)** C4D1 = Cycle 4, Day 1; **(D)** C6D1 = Cycle 6, Day 1. Virtually all the CD34^+^CD38^-^ cells were CD123^+^ and CD33^+^ consistent with AML. The size of this CD123^+^CD33^+^CD34^+^CD38^-^ AML blast population indicated with the arrow in Panel **(A)**, 3^rd^ column, was significantly reduced by APVO436 monotherapy. See also **Table 2**.

We used immunophenotyping by multiparameter flow cytometry to examine the effects of APVO436 on tumor burden reflected by CD123^+^CD34^+^CD38^-^ target blast cells (19). Most of these cells co-express CD33 antigen (i.e., they are CD33^+^CD34^+^CD38^-^) (19). Flow cytometric analysis of post-treatment blood samples from 3 of 4 AML patients (UPN01, UPN03, UPN06) showed decreased numbers of the circulating CD123^+^CD34^+^CD38^-^ and CD33^+^CD34^+^CD38^-^ cells (**Table 2**). The maximum reduction in the numbers of the CD123^+^CD34^+^CD38^-^ cells were 76.1% in UPN01 (C6D1 sample), 57.8% in UPN03 (C1D30 sample), and 99.2% in UPN06 (C4D1 sample). **Figure 1** depicts the multicolor dot profiles from the multi-parameter flow cytometry test that illustrates the rapid and marked depletion of the CD123^+^CD34^+^CD38^-^ and CD33^+^CD34^+^CD38^-^ cells by APVO436 monotherapy in UPN06. Virtually all the CD34^+^CD38^-^ cells were CD123^+^ and CD33^+^ consistent with AML. The size of this CD123^+^CD33^+^CD34^+^CD38^-^ AML blast population indicated with the arrow in Panel A, 3^rd^ column, was significantly reduced by APVO436 monotherapy. In contrast to these 3 AML cases, **Table 2** and **Figure S1** illustrate that the CD34^+^CD38^-^CD123^+^ cells from the 4^th^ AML patient, UPN05, were not depleted to APVO436 treatments, although their expansion was prevented which was associated with stable disease with a time to progression of 238 days. As shown in **Table 2**, reductions of CD123^+^CD34^+^CD38^-^ and CD33^+^CD34^+^ CD38^-^ cells were also observed in post-treatment samples of two MDS patients.

**Table 2:** Pharmacodynamic Effects of APVO436 on Circulating CD123^+^CD34^+^CD38^-^ and CD33^+^CD34^+^CD38^-^ AML Blast Cells.

UPN	CD123^+^CD34^+^CD38^-^Cells Number (% of CD45+)/% Depletion	CD33^+^CD34^+^CD38^-^Cells Number (% of CD45+)/% Depletion	CD45^+^
**UPN01/AML**			
Baseline/C1D1	155 (0.16%)	156 (0.17%)	94415
C2D15	74/52.3%	73/53.2%	86266
C4D1	71/54.2%	72/53.8%	89848
C6D1	37 (0.04%)/76.1%	36 (0.04%)/76.9%	84337
			
**UPN03/AML**			
Baseline/C1D1	2757 (2.8%)	2732 (2.8%)	97928
EOT/C1D30	1163 (1.2%)/57.8%	1153 (1.2%)/57.8%	93827
			
**UPN05/AML**			
Baseline/C1D1	13782 (15.4%)	13147 (14.7%)	89598
C2D15	21115/0%	17185/0%	94131
C4D1	22787/0%	18565/0%	96053
C6D1	23653 (24.3%)/0%	15001 (15.4%)/0%	97346
			
**UPN06/AML**			
Baseline/C1D1	1569 (1.7%)	1552 (1.7%)	92694
C2D15	24/98.5%	23/98.5%	92219
C4D1	12 (0.01%)/99.2%	12 (0.01%)/99.2%	92694
C6D1	33 (0.01%)/97.9%	32 (0.01%)/97.9%	93863
			
**UPN09/MDS**			
Baseline/C1D1	1441 (1.52%)	907 (0.96%	94715
C2D1	1489/0%	1191/0%	94300
C4D1	947/34.3%	575/36.6%	95129
C6D1	1419/4.7%	1421/0%	94893
			
**UPN10/MDS**			
Baseline/C1D1	833 (0.9%)	836 (0.9%)	972155
C2D15	833/0%	775/7.3%	101335
C4D1	477 (0.48%)/42.7%	457/45.8%	99979
EOT/C5D20	2567 (2.5%) /3.1-fold expansion)	2296 (2.3%)/ 2.7-fold expansion	100884

UPN03 had 40% bone marrow blasts at screening and 40% at C1D22. EOT was on C1D30.

UPN10 has 7.5% bone marrow blasts at screening and 20% bone marrow blasts on C5D1. EOT was on C5D20.

These preliminary pharmacodynamic results provided early proof of concept that the CD3xCD123 BiAB APVO436 can activate T-cells and causing depletion of targeted CD123^+^ blast cells in patients with MDS and AML. Notably, at the time of progression, there was a significant expansion of the CD123^+^ population in UPN10 and the cells in UPN03 that were not depleted were CD123^+^. These results illustrate that APVO436 monotherapy failures can occur independent of CD123 expression as the anti-leukemic activity of this BiAB is dependent on the cytotoxic T-cell (CTL) activity of the CD3-expressing T-cell populations that are redirected to CD123^+^ AML/MDS cells.

### Efficacy

**Figure 2:**
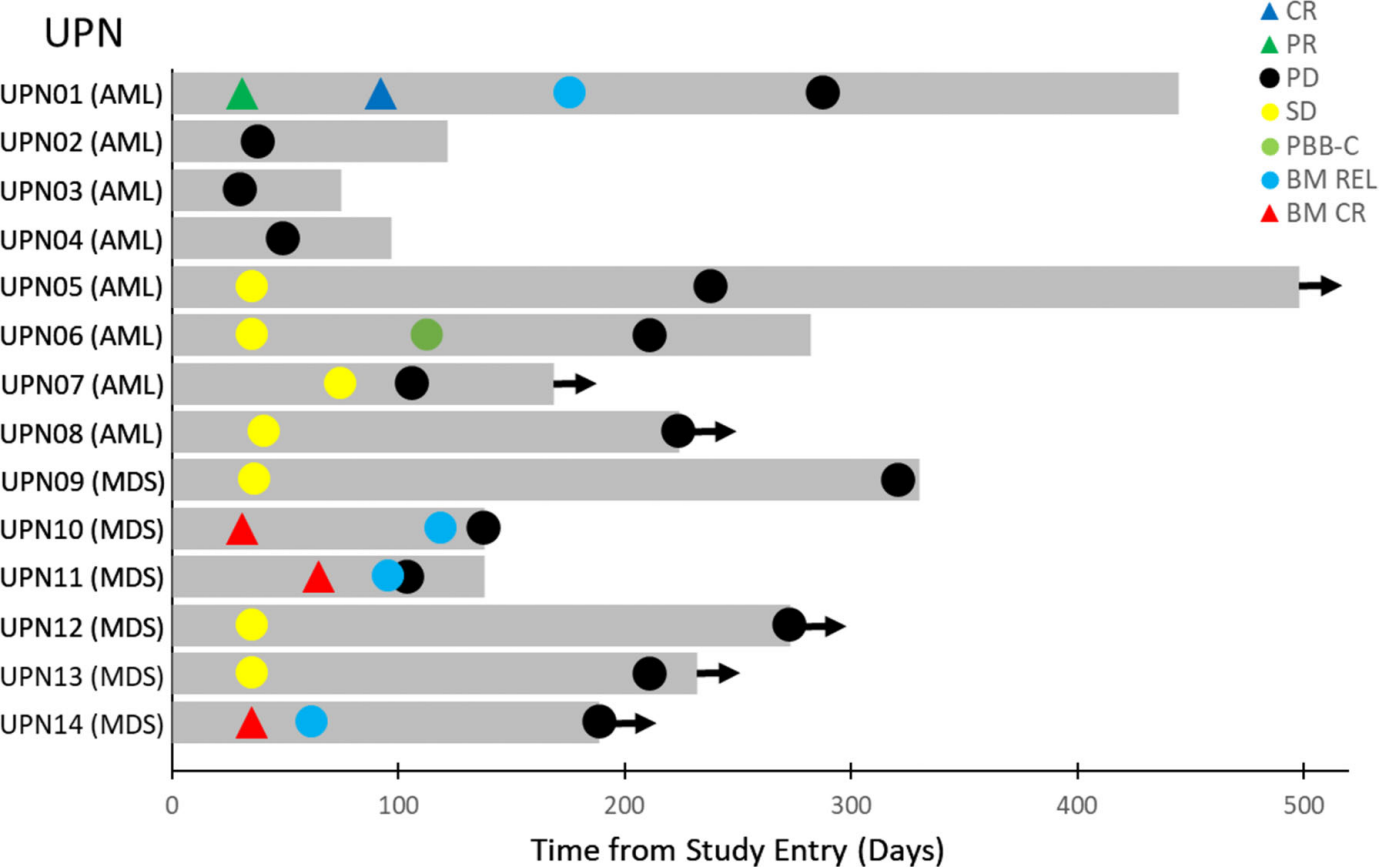
Swimmer Plot of Best Overall Responses. The onset and duration of stable disease (SD, PR, CR, clearance of peripheral blasts, and onset of PD are indicated with specific symbols. Arrow: Alive. The disease progression and survival data were updated since the initial report of the primary study (28).

Of the 8 R/R AML patients, 2 patients (UPN2 and UPN4) had progressive disease (PD) and one had a stable disease (SD)/resistant disease (RES) (UPN3) as best overall response (BOR) and died of leukemia between 75-122 days (**Table 1**). Three patients (UPN05, UPN06, UPN07) had prolonged SD. Their time to progression ranged from 211 days to 238 days. UPN06 from Cohort 7 (Dose level: 0.4 mcg/kg), an AML patient with MDS related features, had a complete clearance of peripheral blasts along with a >50% reduction of the percentage of bone marrow blasts, followed by sustained SD with a time-to-progression of 211 days (**Figure 2**). This patient also had evidence of depletion of target CD123^+^CD34^+^CD38^-^ AML cells (**Table 2** and **Figure 1**). Another AML patient with MDS-related features (UPN01 from Cohort 6B, Dose level: 0.1 mcg/kg), who had 29% bone marrow blasts, unfavorable cytogenetics (del 5q and monosomy 7) and TP53 mutation, whose disease had previously progressed on Venetoclax + Decitabine therapy, achieved a PR at 31 days and CR at 92 days following APVO436 monotherapy with full hematologic recovery as BOR (**Table 1** and **Figure S1**). This patient also had evidence of depletion of target CD123^+^CD34^+^CD38^-^ blast cells (**Table 2**). The onset and duration of the SD, peripheral blood blast count clearance (PBBC-C), PR, or CR in these patients is illustrated by the Swimmer plot depicted in **Figure 2**.

Likewise, 3 MDS patients had SD (50%) and 3 additional MDS patients (50%) had a bone marrow CR at dose levels ranging from 0.1 mcg/kg to 0.4 mcg/kg (**Table 1**): One MDS patient (UPN10 from Cohort 6A, Dose level: 0.2 mcg/kg) had a pretreatment BM blast percentage of 7.5% with 10% cellularity and a C2D1 posttreatment BM blast percentage of 2.4% with 20% cellularity. Another MDS patient (UPN11 from Cohort 7, Dose level: 0.3 mcg/kg) had baseline BM blasts of 11.3% with 20-30% cellularity and a C2D1 posttreatment BM blast percentage of 0% with 50% cellularity. A third MDS patient (UPN12 from Cohort 9, Dose level: 0.4 mcg/kg) had a pretreatment BM blast percentage of 8.2% with 15% marrow cellularity and a C2D1 posttreatment BM showing 2% blasts with 20% cellularity. One MDS patient, UPN09 in Cohort 4, with ASXL-1 mutation showed a decrease in the percentage of myeloblasts in the bone marrow, but this was part of the transformation of the case into a high-risk chronic myelomonocytic leukemia-myeloproliferative neoplasm (CMMN-MPN) with an absolute monocyte count 17,600/µL around Cycle 10 (**Table 1**). The onset of marrow CR and time to progression in these MDS patients is illustrated by the Swimmer plot depicted in **Figure 2**. The median overall survival of the 14 HMA/Venetoclax-resistant AML/MDS patients was 282 days (**Figure S2**).

## Discussion

Therapy-naïve patients with AML who are elderly (>=75) or unfit to receive intensive chemotherapy have high response rates and improved survival when treated with Venetoclax and HMA compared to HMA alone (7, 31). This combination therapy was therefore approved by the Food and Drug Administration (FDA) (15). However, with increasing clinical experience involving the use of Venetoclax, several challenges and limitations of Venetoclax-based therapy have emerged, as emphasized in recent publications (13, 32).

Venetoclax-induced remissions in therapy-naïve older/unfit AML patients are short-lived lasting less than 12 months (median) even after combined use of Venetoclax and HMAs. Furthermore, patients with secondary AML as well as AML patients previously treated with HMAs are less responsive to Venetoclax-based treatment regimens with substantially worse CR rates and <6-month overall survival times (13). Likewise, some AML patient populations in the adverse risk category, such as patients with TP53 mutations, or those with RTK mutations, may exhibit inherent Venetoclax-resistance (13). Unlike its remarkable activity in therapy-naïve AML patients, Venetoclax is not as effective in relapsed AML patients. The reported overall response rate was 21% for relapsed or refractory AML patients treated with venetoclax in combination with HMAs, LDAC, or other agents such as cladribine or midostaurin (33). Therefore, new agents that can potentially be combined with and improve the clinical efficacy of Venetoclax-based treatment regimens for elderly AML patients in the upfront setting are urgently needed.

This analysis provides clinical proof of concept evidence supporting the mechanism of action of APVO436 using the primary data from a recently completed Phase 1B study in R/R AML and MDS patients (28). Reponses in patients resistant to HMA or venetoclax plus HMA included CR in an AML patient with P53/monosomal karyotype and marrow CR in 3 MDS patients, along with 5 AML patients and 6 MDS patients with prolonged stable disease. Median overall survival for all 14 patients was 282 days. Our results should be interpreted with due caution due to the inherent limitations associated with a small size and heterogeneity of the patient population and the fact that prior treatment regimens were not uniform across all patients. One further weakness in our study is the lack of characterization of the patients’ immune repertoire or immunosuppressive bone marrow microenvironment in relationship to the observed clinical effects. In this regard, it will be important to determine if abundance of myeloid-derived suppressor cells (MDSCs) will correlate with resistance to APVO436 as MDSCs have been shown to inhibit the cytotoxic T-cell (CTL) activation by CD3-engaging BiAB (34).

Here we have demonstrated promising early single-agent activity and immunomodulatory effects of APVO436 (given as a weekly IV infusion) in patients who have failed prior treatment with Venetoclax plus HMA for AML or HMA alone for MDS. If confirmed in future studies of APVO436, our observations will add to the growing clinical evidence that BiAB against AML may overcome chemotherapy resistance and thereby improve treatment outcome of patients with adverse cytogenetic features (22, 28). Although single-agent APVO436 has not been associated with dose-limiting myelosuppression, we are planning to formally evaluate in a Phase IB study the tolerability and efficacy of APVO436 in combination with a Venetoclax plus Azacitidine backbone in adverse-risk fit AML patients (NCT03647800, Expansion phase, Cohort 2). There will be a 2-week lead-in phase of Venetoclax plus Azacitidine to mitigate risk of TLS and cytokine release syndrome (CRS). We hypothesize that the addition of APVO436 will eradicate residual CD123^+^ blasts as well as leukemic stem cells that are resistant to Venetoclax, leading to more durable responses. Likewise, Venetoclax is currently being studied in combination with other CD123-targeted therapies such as SL-401 and IMGN632 [ClinicalTrials.gov identifiers: NCT03113643, NCT04086264]. It is noteworthy that Venetoclax augments T-cell effector function by increased production of reactive oxygen species (35). Likewise, Azacitidine enhances sensitivity of AML cells to cytotoxic T-cells *via* activation of the STING pathway (35). Therefore, a combination of T-cell redirecting BiAb such as APVO436 with Venetoclax or Azacitidine may have clinical potential.

The results reported here informed the design of currently accruing Cohort 2 of the Expansion phase of the Phase 1B study (NCT03647800). In future studies of APVO436, it will be important to prospectively evaluate the dynamic changes of the pharmacodynamic parameters, including target AML blast populations as well as levels of T-cell derived cytokines and correlate such changes with induction of remission as well as treatment-emergent adverse events (e.g. CRS, infusion related reactions, neurotoxicity).

## Data Availability Statement

The original contributions presented in the study are included in the article/**Supplementary Material**. Further inquiries can be directed to the corresponding author.

## Ethics Statement

The studies involving human participants were reviewed and approved by Central IRB (WCG; IRB00000533). The patients/participants provided their written informed consent to participate in this study.

## Author Contributions

All authors have equally contributed to this manuscript. TL, AM, PP, PS, EC, CRC, EW, and JW assisted in study design, served as site investigators, screened and recruited participants, administered treatments, assessed adverse events and disease responses, collected safety and efficacy data, met regularly to review study data during the study, and reviewed the manuscript. FU designed the evaluations reported in this paper, directed the data compilation and analysis, and prepared the initial draft of the manuscript. FU, AS, and CL analyzed and validated data, performed statistical analyses. Each author reviewed and revised the manuscript, and provided final approval for submission of the final version.

## Funding

This study received funding from Aptevo Therapeutics. The funder had the following involvement with the study: 1. Provided funding to a clinical research organization for the operationalization of the study by clinical monitoring, medical monitoring, pharmacovigilance, and data management; 2. Provided site awards to the investigative sites to compensate the sites for their clinical research expenses; and 3. Sponsored the IND, funded the regulatory affairs and quality assurance activities to ensure ICH/GCP compliance. The sponsor did not participate in the safety or efficacy assessments of the investigators.

## Conflict of Interest

Author FU is employed by Ares Pharmaceuticals, he served as a consultant for Aptevo Therapeutics and he serves as a consultant to Reven Pharmaceuticals. Author CL is employed by Oncotelic Therapeutics, she served as a consultant to Aptevo Therapeutics and she serves as a consultant to Reven Pharmaceuticals. Author AS also served as a consultant to Aptevo.

The remaining authors declare that the research was conducted in the absence of any commercial or financial relationships that could be construed as a potential conflict of interest.

## Publisher’s Note

All claims expressed in this article are solely those of the authors and do not necessarily represent those of their affiliated organizations, or those of the publisher, the editors and the reviewers. Any product that may be evaluated in this article, or claim that may be made by its manufacturer, is not guaranteed or endorsed by the publisher.

## Supplementary Material

Supplementary Figure 1 Circulating AML-LSC Cells in VENAZA-resistant Relapsed AML Patient Receiving APVO436 Monotherapy. Virtually all the CD34^+^CD38^-^ cells were CD123^+^ and CD33^+^ consistent with AML. The size of this CD123^+^CD33^+^CD34^+^CD38^-^ AML LSC population indicated with the arrow in the 3^rd^ column, did not significantly change during APVO436 monotherapy. See also **Table 2** in the main manuscript.

Supplementary Figure 2 Survival Outcome of R/R AML/MDS Patients According to Prior Therapy. Depicted is the overall survival curve of the 14 HMA/Venetoclax-resistant AML/MDS patients shown in **Figure 2** in the main manuscript. All patients were treated with APVO436 monotherapy.
