# Supplementary material for: Post-hoc Analysis of Pharmacodynamics and Single-Agent Activity of CD3xCD123 Bispecific Antibody APVO436 in Relapsed/Refractory AML and MDS Resistant to HMA or Venetoclax Plus HMA
Source: Front Oncol. 2022 Jan 13;11:806243. doi: 10.3389/fonc.2021.806243 (PMC8793782; doi:10.3389/fonc.2021.806243)
Supplement: Supplementary Figure 1 — Circulating AML-LSC Cells in VENAZA-resistant Relapsed AML Patient Receiving APVO436 Monotherapy. Virtually all the CD34+CD38- cells were CD123+ and CD33+ consistent with AML. The size of this CD123+CD33+CD34+CD38- AML LSC population indicated with the arrow in the 3rd column, did not significantly change during APVO436 monotherapy. See also Table 2 in the main manuscript. [file DataSheet_1.docx]

**SUPPLEMENTAL MATERIAL**

**Post-hoc Analysis of Pharmacodynamics and Single-agent Activity of CD3xCD123 Bispecific Antibody APVO436 in Relapsed/Refractory AML and MDS Resistant to HMA or Venetoclax Plus HMA**

Justin Watts^1^, Tara L. Lin^2^, Alice Mims^3^, Prapti Patel^4^, Cynthia Lee^5^, Anoush Shahidzadeh^5^, Paul Shami^6^, Elizabeth Cull^7^, Christopher R. Cogle^8^, Eunice Wang^9^, Fatih M. Uckun^5,10^

^1^University of Miami Sylvester Comprehensive Cancer Center, Miami, Florida 33136, USA ^2^University of Kansas Cancer Center and Medical Pavillon, Westwood, Kansas 66205, USA; ^3^The Ohio State University Wexner Medical Center/James Cancer Hospital, Columbus, Ohio, United States, 43210;    ^4^University of Texas Southwestern Medical Center Dallas, Texas, United States, 75390;  ^5^Aptevo Therapeutics, Seattle, WA 98121; ^6^University of Utah, Huntsman Cancer Institute Salt Lake City, Utah, United States, 84112;   ^7^Greenville Health System, Institute for Translational Oncology Research, Greenville, South Carolina, United States, 29605;  ^8^University of Florida, Gainesville, Florida 32610, USA; ^9^Roswell Park Cancer Institute; Buffalo, New York, United States, 14263; ^10^Immuno-Oncology Program, Ares Pharmaceuticals, St. Paul, MN 55110, USA

**
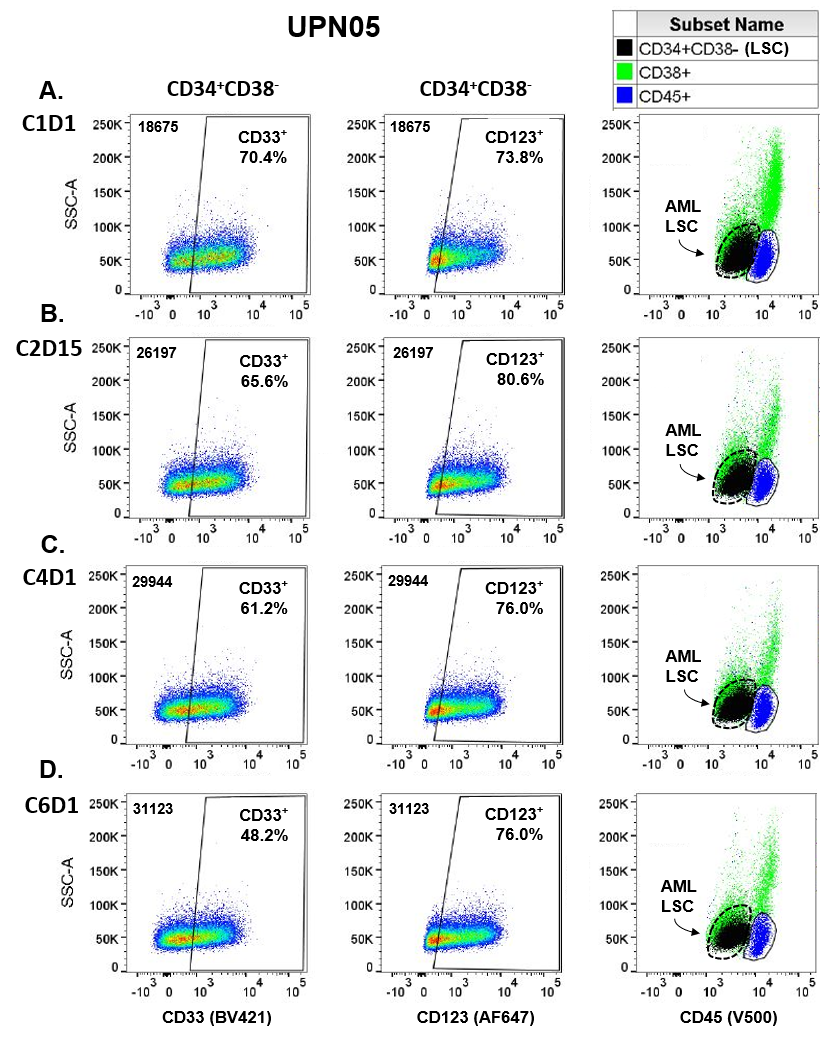
SUPPLEMENTAL FIGURES**

**Figure S1. Circulating AML-LSC Cells in VENAZA-resistant Relapsed AML Patient Receiving APVO436 Monotherapy.** Virtually all the CD34^+^CD38^-^ cells were CD123^+^ and CD33^+^ consistent with AML. The size of this CD123^+^CD33^+^CD34^+^CD38^-^ AML LSC population indicated with the arrow in the 3^rd^ column, did not significantly change during APVO436 monotherapy. See also Table 2 in the main manuscript.


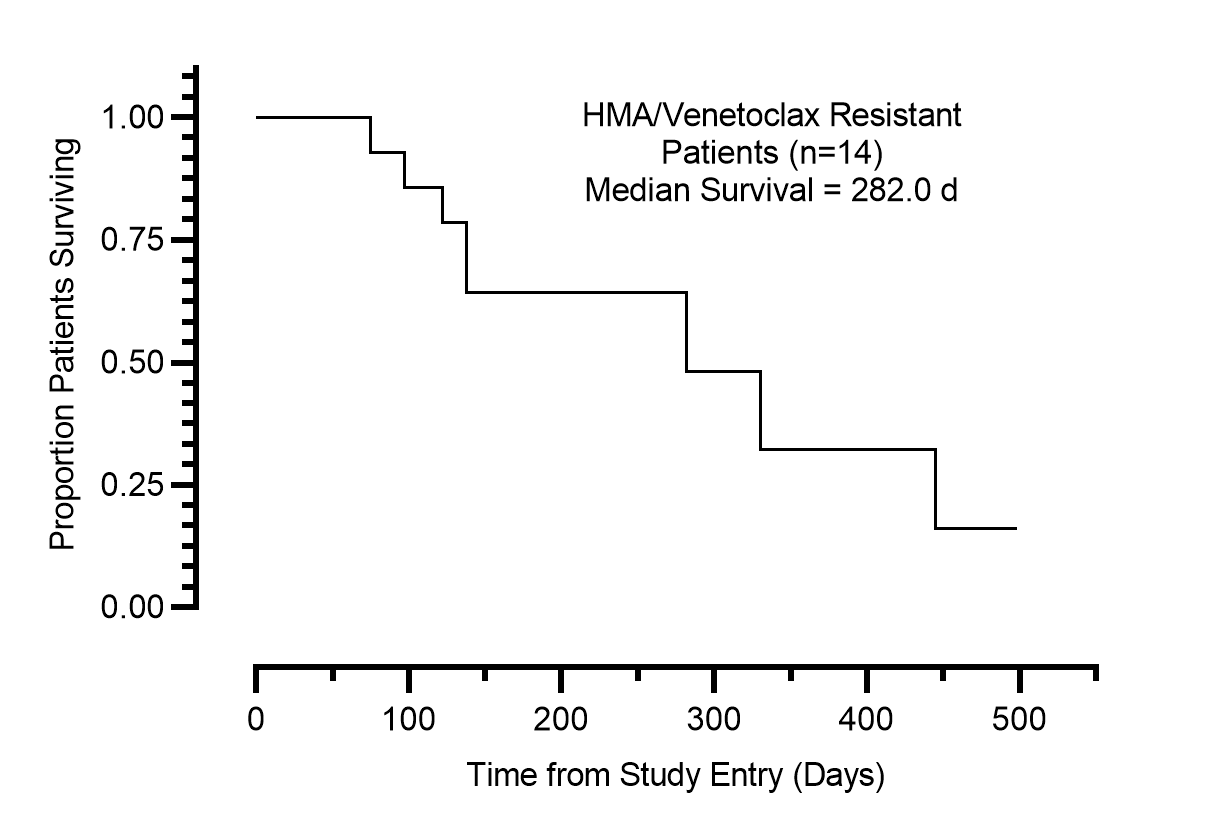


**Figure S2.** **Survival Outcome of R/R AML/MDS Patients According to Prior Therapy.** Depicted is the overall survival curve of the 14 HMA/Venetoclax-resistant AML/MDS patients shown in Figure 2 in the main manuscript. All patients were treated with APVO436 monotherapy.
